# Central Venous Catheter Adverse Events Are not Associated with Crowding Indicators

**DOI:** 10.5811/westjem.2020.10.48279

**Published:** 2021-01-20

**Authors:** Daniel L. Theodoro, Niraj Vyas, Enyo Ablordeppey, Brian Bausano, Stephanie Charshafian, Phillip Asaro, Richard T. Griffey

**Affiliations:** * Washington University School of Medicine, Department of Emergency Medicine, St. Louis, Missouri; † University of Pennsylvania, Perelman School of Medicine, Penn Acute Research Collaboration (PARC), Philadelphia, Pennsylvania

## Abstract

**Objective:**

Crowding in the emergency department (ED) impacts a number of important quality and safety metrics. We studied ED crowding measures associated with adverse events (AE) resulting from central venous catheters (CVC) inserted in the ED, as well as the relationship between crowding and the frequency of CVC insertions in an ED cohort admitted to the intensive care unit (ICU).

**Methods:**

We conducted a retrospective observational study from 2008–2010 in an academic tertiary care center. Participants undergoing CVC in the ED or admitted to an ICU were categorized by quartile based on the following: National Emergency Department Overcrowding Scale (NEDOCS); waiting room patients (WR); ED patients awaiting inpatient beds (boarders); and ED occupancy (EDO). Main outcomes were the occurrence of an AE during CVC insertion in the ED, and deferred procedures assessed by frequency of CVC insertions in ED patients admitted to the ICU.

**Results:**

Of 2,284 ED patients who had a CVC inserted, 293 (13%) suffered an AE. There was no association between AEs from ED CVCs and crowding scales when comparing the highest crowding level or quartile to all other quartiles: NEDOCS (dangerous crowding [13.1%] vs other levels [13.0%], P = 0.98); number of WR patients (14.0% vs 12.7%, P = 0.81); EDO (13.0% vs 12.9%, P = 0.99); and number of boarding patients (12.0% vs 13.3%), P = 0.21). In a cohort of ED patients admitted to the ICU, there was no association between CVC placement rates in the ED and crowding scales comparing the highest vs all other quartiles: NEDOCS (dangerous crowding 16% vs all others 16%, P = 0.97); WR patients (16% vs 16%, P = 0.82), EDO (15% vs. 17%, P = 0.15); and number of boarding patients (17% vs 16%, P = 0.08).

**Conclusion:**

In a large, academic tertiary-care center, frequency of CVC insertion in the ED and related AEs were not associated with measures of crowding. These findings add to the evidence that the negative effects of crowding, which impact all ED patients and measures of ED performance, are less likely to impair the delivery of prioritized time-critical interventions.

## INTRODUCTION

Emergency department (ED) crowding is defined as the environment in which local demand for emergency care outpaces available resources. Crowding is associated with delays in care and poor outcomes. Crowded EDs delay antibiotic and analgesic delivery. Crowding delays damage control resuscitation in major trauma; additionally, patients admitted on days with greater ambulance diversion, a measure of high ED crowding, experience increased hospital lengths of stay, costs, and mortality.^1^–^8^ Conceptually, crowding can cause providers to deliver hurried care and miss critical steps during complex procedures.^9^

Placement of a CVC is a complex, multi-step procedure that requires equipment, operator assistance, and time to complete. Ultrasound guidance, training, and patient comorbidities all influence success or failure of CVC placement; however, the role that crowding may play on procedure success is not known. Describing the association between crowding and the safety of CVC insertion in the ED is important because this impacts decision-making related to staffing, guidelines, and equipment. We hypothesized that the effects of crowding may impact emergency physician (EP) performance during CVC placement or may prompt EPs to defer the procedure to downstream providers. Therefore, the objective of this study was to examine the association between measures of crowding and frequency of AEs during CVC insertion in the ED and study the relationship between crowding and the frequency of CVC insertion among ED patients admitted to the ICU.

## METHODS

### Study Setting and Population

We conducted a retrospective observational study from March 7, 2007–July 31, 2010 from an urban academic adult-only ED Level I trauma center with an annual census of 95,000 visits. Any subject older than 18 years of age who underwent CVC placement in the ED was eligible for the study. To estimate whether ED CVC placement was deferred, we included for analysis a second sample of patients admitted from the ED to any intensive care unit (ICU) of the hospital during the identical time frame. In this second subset of patients destined for ICU admission, subjects were identified as recipient or non-recipient of a CVC placed in the ED. The study was approved by the Human Research Protection Office (HRPO) of Washington University School of Medicine.

### Study Protocol and Measurements

To obtain data on CVC inserted in the ED, we created a standardized and partly auto-populated procedure note in our electronic health record (EHR). This included details about the time CVC insertion took place, its anatomic location, the method of insertion, use of ultrasound, operator, and any immediate adverse events (AE). We held quarterly educational sessions during the first year of standardized note implementation that included the definitions of AEs and their documentation. This was followed with an audit and feedback process to ensure data capture and fidelity. Operator skill was based upon the number of CVCs performed during the operator’s career, which was initially self-reported and then updated based on our database. We grouped skill level as 20 or fewer, 20–50, and more than 50 CVCs performed. We abstracted patient level data including age and diagnoses from the health record as assigned by the EP at the time of the visit.

**Population Health Research Capsule**

What do we already know about this issue?

High levels of Emergency Department (ED) crowding delay ED operations and increase hospital length of stay, cost of care, and patient mortality.

What was the research question?

Does high-crowding increase the rate of complications from central venous catheters inserted in the ED?

What was the major finding of the study?

We found no association between crowding and adverse events stemming from ED central venous catheter insertions.

How does this improve population health?

The negative impact of crowding does not impair the delivery and outcome of time-critical procedures, but will affect ED performance and patients in other ways.

The Division’s Information Technology node collected data independently to describe operational data. We queried our research copy of the ED EHR for data elements needed to estimate the level of crowding at the time of CVC placement. This dataset receives and stores updates at 15-minute intervals throughout the day. We collected information on the number of patients in the waiting room (WR), the number of admitted patients in the ED awaiting inpatient beds or “boarders,” and ED occupancy (EDO) because these measures of crowding have been linked to the quality of care or have been validated.^2^,^10^ EDO was defined as the total number of patients in the ED divided by the total number of ED beds. Visit data were also used to generate a National Emergency Department Overcrowding Scale (NEDOCS) score that uses several operational variables to categorize different levels of crowding ranging from “not busy, 0–20”; “busy, 21–60”; “extremely busy but not overcrowded, 61–100”; “overcrowded; 101–140”, “severely overcrowded; 141–180”; and “dangerously overcrowded >180.”^11^

We hypothesized that EPs may also defer CVC placement in the ED during moments of high crowding. To obtain data regarding this possibility, we created a separate dataset of ED patients admitted to any hospital ICU during the same months of our original data set. Patients in the ICU present with greater comorbidity and greater intensity of illness and are most likely to require ED CVC insertion.^12^–^14^ Admission to the ICU was defined according to the documented destination in the EHR. In this dataset we determined CVC insertion by procedure notes collected as described above, and we collected measures of crowding in the same manner.

### Outcome Measures

Our primary outcome was AEs attributed to CVCs inserted in the ED. AEs were defined as a failed first-pass attempt ultimately requiring a secondary attempt (rescue); unsuccessful insertion (procedure aborted); bleeding; hematoma; arterial puncture; and pneumothorax. Pneumothoraces were identified immediately after insertions of catheters to the chest and neck by chest radiograph (CXR). Trained research assistants, blinded to the main outcome of the study, also performed a retrospective review of serial CXRs for 48 hours post insertion to identify latent pneumothorax not identified in the ED. Training consisted of primary investigator educational sessions. Reviewers were blinded to the main outcome of the study. A small subset of records was oversampled to determine inter-rater reliability.

Secondary outcomes included the association between ultrasound utilization, level of operator performing the procedure, central line-associated blood stream infections (CLABSI) attributed to ED CVC placement and their association to levels of crowding. The number of ED CLABSIs was obtained from infection control. This data was limited to 28 months of the total 41-month study (March 7, 2008–June 1, 2010) and is reported separately.

Lastly, we measured the frequency of CVCs placed in the ED among a subset of all ED patients admitted to the ICU. The frequency of ED CVC in this subset was compared to different levels of crowding to examine the possibility that EPs may defer the procedure in the ED during busier times.

### Statistical Analysis

We hypothesized that AEs would occur with more frequency in dangerous or severe crowding conditions according to the NEDOCS score compared to all other levels of crowding. We determined we would need to collect 2200 ED CVC insertions to achieve 80% power to observe a 5% difference in AEs during these levels of crowding.^15^,^16^

Parametric data are presented as means ± standard deviations (SD), and non-parametric data are expressed as interquartile ranges (IQR). Waiting room patients and boarders were categorized by quartiles and assessed by highest quartile vs the lowest quartiles, as well as highest compared to all others.^1^ ED occupancy (percentage of overall ED beds filled) was categorized as all beds occupied vs any open beds. We analyzed the NEDOCS score by a five-category analysis consisting of the standard NEDOCS categorization with the lowest two rankings combined and by categorizing the top two NEDOCS scores (severe or dangerous crowding) vs the remaining lower scores.^17^,^18^ We used *R*, v 3.6.2 (*R* Foundation, Vienna, Austria) to perform chi-square and Fisher’s exact tests to assess the differences between groups of categorical data. We used logistic regression to control for variables previously demonstrated to affect AE rates when evaluating potential relationships with measures of crowding including renal failure, physician experience, and ultrasound guidance. We used the le Cessie-vanHouwelingen-Copas-Hosmer unweighted sum of squares to test for global goodness of fit (GoF).

## RESULTS

During the study period 2284 subjects underwent CVC in the ED. The mean age was 59 years (±24 years). Emergency department diagnoses at the time of admission were as follows: infectious 728 (32%); metabolic 311 (14%); cardiac 299 (13%); trauma 177 (8%); and other 769 (34%), The mean ED NEDOCS score was 117.6 (SD ± 43.9) (crowded), and the mean number of WR patients was 15 (SD ± 11.5). The mean number of patients awaiting beds (number of boarding patients) was 9 (SD ± 5) and the EDO median was 100% (IQR = 91% – 100%). The least experienced operators placed 608 CVCs (27%) while the most experienced placed 568 CVCs (25%). Operators used ultrasound assistance to place CVCs in 1392 (61%) insertions. Adverse events occurred in 297 (13%) attempts (Table 1). The most common AE was failed first-pass attempt requiring rescue.

**Figure 1:**
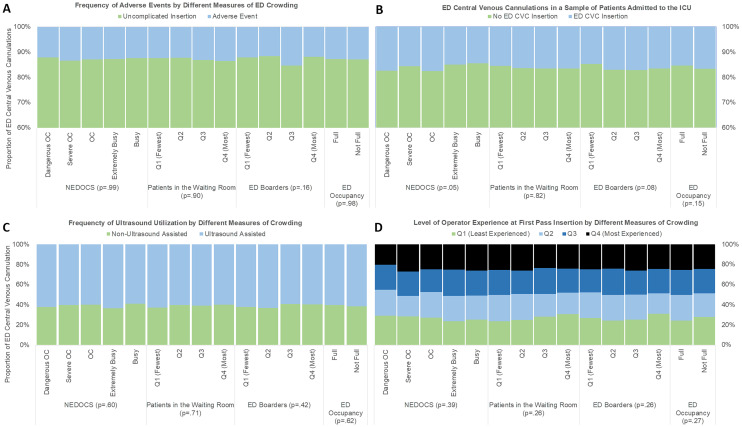
Outcomes and characteristics of central venous cannulations performed in the emergency department by different scales of crowding. *NEDOCS*, National Emergency Department Overcrowding Scale; *ED*, emergency department; *OC*, overcrowding; *Q*, quartile.

The ED was dangerously crowded during 30.4% of CVC insertions. A total of 91 (13.1%) AEs occurred while the ED was dangerously crowded compared to 206 (13.0%), *P* = 0.98, at all other levels of crowding. The number of AEs during CVC insertion when the WR was most full was 68 (14.0%) compared to 219 (12.7%), P = 0.81, during all other times. The number of AEs during highest EDO was 202 (13.0%) compared to 95 (12.9%), P = 1.00, at all other times. When the ED held the greatest number of boarded patients the number of AEs during CVC insertion in the ED was 60 (12.0%) compared to 236 (13.3%), *P* = 0.21, when the ED held fewer boarded patients. Figure 1 panel A demonstrates the association between AEs and different levels of crowding scales by quartile. There was no association between measures of crowding scale by quartile and AEs.

ED ultrasound utilization and level of operator experience during CVC insertion did not vary by measure of crowding (Figure 1, Panel C and Panel D). There was no association between CLABSI and levels of crowding (Table 2).

Table 3 demonstrates the results of a logistic regression model including known risk factors for CVC AEs (ultrasound utilization and renal disease) and different levels of crowding. Fewer AEs were associated with ultrasound-assisted CVC placement, but there was no effect of ED crowding in any of our models. Global GoF tests indicate that all models are an appropriate fit.

### Deferred Procedures

A total of 9241 patients were admitted to the ICU during this time period, and 1497 (16.2%) underwent CVC placement in the ED. The mean age was 58 years (SD ± 18.9). Emergency department diagnoses at the time of admission were as follows: other 3431 (37%); trauma 2610 (28%); infectious 1405 (15%); and cardiac 1,011 (11%). Mean measures of crowding were as follows: NEDOCS, 123.7 (SD ± 43.5); number of waiting room patients, 16 (SD ± 11.6); EDO median = 100% (IQR 91% – 100%); and number of ED boarding patients, 10 (SD ± 5).

The frequency of ED CVC placement during severe or dangerous crowding was 16% (540 patients) and 16% (957 patients) (*P* = 0.98) during lower levels of crowding. There was no association between ED CVC placement and other scores of crowding comparing the highest vs all other quartiles: WR patients (16% vs 16%, *P* = 0.82), EDO (15% v. 17%, *P* = 0.15): number of boarding patients (17% vs 16%, *P* = 0.08). Figure 1 Panel B shows the frequency of CVC insertions in the ED occurring during different levels of crowding. There was no association between frequency of ED CVC insertions and crowding level by quartile.

## DISCUSSION

Our study found no association between measures of high ED crowding and CVC AEs. Conceptually, crowding has an effect on ED “throughput,” interfering with normal operations and possibly impacting physician performance. We hypothesized that physicians may feel hurried, causing an increase in the risk of skill-set and task-based error during periods of excessive ED crowding.^19^ This cognitive strain may interfere with performing a moderately complex procedure resulting in greater AEs. Providers may skip essential protective steps that result in greater risk of harm. For example, providers may skip placing the patient in Trendelenburg position or avoid using ultrasound. However, our results suggest that AEs occur with similar frequencies during all levels of ED crowding. We did observe the protective effect of ultrasound, which may have contributed to our findings. Ultrasound-guided CVC is well documented to decrease the risk of AEs in the ED setting and may mitigate some risk during times of excessive crowding by decreasing the complexity of the procedure.^20^,^21^ Crowding had no effect on the level of training of the physician performing the procedure or whether CVCs were placed with ultrasound guidance, again suggesting that physicians treat critically ill patients similarly during different periods of ED crowding.

We also found that EPs were as likely to insert CVCs in critically ill patients during times of crowding as when the ED was less crowded, suggesting that CVC placement in the ED is not deferred to downstream clinical services. We hypothesized that EPs would perform CVC with less frequency during periods of high ED crowding since performing the procedure requires an investment of time that is otherwise not focused on other patients. We found that this relationship did not occur in a population of ED patients admitted to the ICU suggesting that EPs do not delegate critical procedures to downstream healthcare providers. Our findings agree with those of Jo et al who found that critical procedures, including CVC placement, were not delayed with the exception of a subset of trauma patients at the busiest quartiles.^22^ Wu et al, noting coagulation reversal procedures occurred less often during high levels of crowding among trauma patients, suggested that crowding caused CVC insertion delays; however, the authors did not report the specific data.^1^ Our study was not designed to examine the effect of CVCs placed in a subset of trauma patients. However, trauma patients represented the majority of patients in our sample of patients destined to the ICU and we did not find any association.

Crowding may not affect all patients similarly. Harris et al suggests crowding affects patients of variable acuity differently.^23^ Crowding may not impact those sick enough to “skip the line.”^24^ Patients with acute stroke symptoms do not experience delays in care during periods of crowding, and crowding may not cause clinically important delays for patients requiring emergent percutaneous coronary thrombolytic angioplasty.^23^ Likewise, the mortality and quality of resuscitation among cases of out-of-hospital cardiac arrest does not differ by measure of crowding.^25^ Crowding affects those triaged in the highest, most acute category the least.^26^ Our data support this argument and suggests that crowding may not exert a direct effect on the outcomes of CVCs placed in ED patients, the majority of whom are critically ill.

Crowding may not factor into the physician’s interaction with a patient who is critically ill. Crowding affects systems-based interactions. For example, in a cohort of patients with pneumonia, Fee et al found crowding caused delays in tasks that required nursing (antibiotic delivery) and system-based tasks (CXR results from radiology reporting) but not physician-level tasks (antibiotic ordering).^27^ While Peltan et al and Gaieski et al observed an increased in time to administration of antibiotics and intravenous fluids among septic patients, they did not capture whether these were physician or nursing delays.^7^,^28^ Owyang et al noted departures from lung protective strategies among ED patients on ventilators as the ED became busier, requiring combined respiratory therapist and physician bedside attention.^29^ Asaro et al suggested that physician treatment time is most strongly influenced by clinical and demographic factors, not crowding measures.^30^ This suggests that crowding may exert its greatest effect when less than critically ill patients rely on systemic efficiencies to achieve high-quality care.

Crowding’s greatest impact may be felt by less obviously sick patients. For example, acute stroke evaluation is not delayed by crowding, but patients with subtle symptoms do experience delays to CT imaging.^31^,^32^ Similarly, crowding can cause lab delays in obtaining critical troponin levels in non-ST elevation myocardial infarction (STEMI) cases while STEMI cases proceed with little pause to invasive interventions. We did not capture the effect on other processes occurring in parallel during delivery of intense ED care, and here is where others have found meaningful delays. Our theoretical model did not address these relationships that may have more indirect effect on overall ED quality of care. We found no evidence of a direct relationship between crowding and AEs from CVCs inserted in the ED.

## LIMITATIONS

This study was a single-center study thereby limiting the generalizability of the results, a central limitation to our conclusions. Additionally, we used a composite AE because we could not use a specific CVC AE to power our study. All AEs are not equivalent—clearly a pneumothorax is not equivalent to a failed attempt; and a “rescue attempt” of a novice is not identical to one required by an expert. This reduced our ability to identify serious AEs that may have a closer relationship to ED crowding measures.

Our retrospective review relied on self-report of AEs following CVC insertion. Although we did not encounter discrepancies between reported AEs and our patient safety officer, it is possible that minor AEs went under-reported, thus lowering the probability of finding an association between crowding and CVC AEs. The fluidity of crowding makes for measurement challenges. While we linked procedure time documentation to crowding measures, it is possible that the procedure took place when crowding scores were slightly different. It is therefore possible that some AEs took place during different measures of crowding categories. Lastly, staffing has been proposed in some studies to play a mediating role in crowding’s impact on outcomes in stroke patients.^31^ In our study, differences in ED staffing were not specifically accounted for and may have played a role in procedural outcomes.

Our data are retrospective and over 10 years old because our protocol encompassed a unique time frame in which sepsis care encouraged high rates of CVC insertions in the ED, CLABSI data, CVC safety data, and crowding metrics were systematically and simultaneously collected before they were disrupted by a system-wide adoption of a new EHR. Care patterns may now differ, especially in cases of sepsis. Rather than re-collecting new data, we elected to evaluate the available retrospective, albeit older, data. We propose that performance of CVC insertions has changed little if at all during this time frame making it unlikely that our data misrepresent current clinical practice.

## CONCLUSION

In a large, academic tertiary-care center, frequency of CVC insertion and related AEs are not associated with measures of crowding. These findings add to the evidence that the negative effects of crowding, which impact all ED patients and measures of ED performance, are less likely to impair the delivery of prioritized time-critical interventions.

## Supplementary Information



## Figures and Tables

**Table 1:** Adverse event during emergency department central venous cannulation by type.

	Adverse Event		No Adverse Event	

	n	(%)	n	(%)
All adverse events	293	(13)	1,991	(87.1)
Adverse event by type				
Failed first-pass attempt requiring rescue	224	(9.8)	2,060	(90.2)
Aborted procedure	3	(.1)	2,281	(99.9)
Hematoma	38	(1.7)	2,246	(98.3)
Arterial puncture	15	(.7)	2,269	(99.3)
Pneumothorax*	13	(.6)	2,271	(99.4)

* Kappa results for the retrospective chart review of pnuemothoraces was 0.99.

**Table 2:** Association between central line-associated-blood stream infections and crowding measures.

Crowding Measure	CLABSI N = 10	No CLABSI N = 1533	P-Value*
NEDOCS			
Dangerous crowding	1 (10%)	98 (6%)	0.84
Severe crowding	2 (20%)	311 (20 %)	
Crowding	4 (40%)	540 (35 %)	
Extremely busy, no crowding	2 (20%)	445 (30 %)	
Busy	1 (10%)	139 (9 %)	
Patients in the waiting room			
Q1 (fewest)	2 (20 %)	458 (30%)	0.43
Q2	3 (30 %)	431 (28%)	
Q3	1 (10 %)	342 (22 %)	
Q4 (most)	4 (40 %)	302 (20%)	
ED Occupancy			
Full	7 (70%)	1,045 (68%)	1.0
Not full	3 (30%)	488 (32 %)	
# of ED patients awaiting inpatient beds			
Q1 (fewest)	3 (30%)	379 (25%)	0.97
Q2	3 (30%)	404 (26%)	
Q3	2 (30%)	395 (26%)	
Q4 (most)	2 (30%)	355 (23%)	

* Fisher’s exact test.

*CLASBI*, central line-associated blood stream infections; *NEDCOS*, National Emergency Department Overcrowding Scale; *Q*, quartile; *ED*, Emergency Department.

**Table 3:** Adjusted odds ratio for likelihood of adverse event during central venous catheter insertions in the emergency department.

Variable	OR (95% CI)	OR p-value	GoF P-value
AE ~ NEDOCS + US + Renal + Exp			0.29
Dangerously crowded	0.98 (0.56 – 1.71)	0.93	
Severely crowded	1.11 (0.69 – 1.82)	0.69	
Crowded	1.04 (0.66 – 1.67)	0.87	
Extremely busy, not crowded	1.06 (0.67 – 1.72)	0.81	
Busy	–––	–––	
AE ~ Waiting + US + Renal + Exp			0.32
Highest quartile	1.10 (0.77 – 1.58)	0.61	
3rd quartile	1.06 (0.74 – 1.51)	0.76	
2nd quartile	0.97 (0.69 – 1.38)	0.88	
1st quartile	–––	–––	
AE ~ Beds + US + Renal + Exp			0.27
Full occupancy	0.99 (0.77 – 1.30)	0.96	
Not at full occupancy	–––	–––	
AE ~ Boarding + US + Renal + Exp			0.91
Highest quartile	0.97 (0.68 – 1.38)	0.85	
3rd quartile	1.32 (0.95 – 1.85)	0.10	
2nd quartile	0.97 (0.67 – 1.38)	0.92	
1st quartile	–––	–––	
AE ~ US			1.00
Ultrasound assisted	0.69 (0.54 – 0.88)	0.003	
AE ~ Exp + US			0.46
Highest Quartile	0.91 (0.65 – 1.28)	0.59	
3rd quartile	0.74 (0.52 – 1.06)	0.10	
2nd quartile	1.08 (0.77 – 1.50)	0.66	
1st quartile	–––	–––	
AE ~ Renal + US			0.08
Renal disease	0.87 (0.59 – 1.24)	0.44	

*NEDOCS*, National Emergency Department Overcrowding Scale; *OR*; odds ratio; *GoF*, goodness of fit; *AE*, adverse events; *US*, ultrasound; *Exp*, experience.
