# Supplementary material for: Central Venous Catheter Adverse Events Are not Associated with Crowding Indicators
Source: West J Emerg Med. 2021 Jan 20;22(2):427–34. doi: 10.5811/westjem.2020.10.48279 (PMC7972355; doi:10.5811/westjem.2020.10.48279)
Supplement: Supplementary file 1 [file wjem-22-427-s001.pdf]

Crowding Measure	NO AE<br>1991 (87.17%)	AE<br>293 (12.83%)	P Value
NEDOCS			0.9898
Dangerous OC	218 (10.9%)	30 (10.2%)	
Severe OC	387 (19.4%)	60 (20.5%)	
OC	650 (32.6%)	96 (32.8%)	
Extremely Busy, no OC	545 (27.4%)	80 (27.3%)	
Busy	191 (9.6%)	27 (9.2%)	
Patients in the WR			0.9034
Q1	508 (26.4%)	72 (25.4%)	
Q2	549 (28.5%)	77 (27.2%)	
Q3	450 (23.4%)	68 (24.0%)	
Q4	418 (21.7%)	66 (23.3%)	
ED Occupancy			0.9774
Full	1351 (67.9%)	198 (67.6%)	
Not Full	640 (32.1%)	95 (32.4%)	
# of ED Patients Awaiting Inpatient Beds			0.1570
Q1	502 (25.3%)	69 (23.6%)	
Q2	519 (26.2%)	68 (23.3%)	
Q3	521 (26.2%)	95 (32.5%)	
Q4	445 (22.4%)	60 (20.5%)	
Ultrasound Use			0.003*
With Ultrasound	1237 (62.1%)	155 (52.9%)	
Without Ultrasound	754 (37.9%)	138 (47.1%)	
Renal Disease			0.3548
With Renal Disease	296 (14.9%)	37 (12.6%)	
Without Renal Disease	1695 (85.1%)	256 (87.4%)	

Association Between Adverse Events During Emergency Department Central Venous Cannulation and Known Risk Factors

Crowding Measure	Non-Ultrasound Assisted 892 (39.1%)	Ultrasound Assisted 1392 (60.9%)	P Value
NEDOCS			
Dangerous OC	94 (10.5%)	154 (11.1%)	0.6039
Severe OC	179 (20.1%)	268 (19.3%)	
OC	300 (33.6%)	446 (32.0%)	
Extremely Busy, no OC	229 (25.7%)	396 (28.4%)	
Busy	90 (10.1%)	128 (9.2%)	
Patients in the WR			
Q1	215 (24.9%)	365 (27.1%)	0.7093
Q2	250 (28.9%)	376 (27.9%)	
Q3	203 (23.5%)	315 (23.4%)	
Q4	194 (22.5%)	290 (21.5%)	
ED Occupancy			
Full	293 (32.8%)	442 (31.8%)	0.6168
Not Full	599 (67.2%)	950 (68.2%)	
# of ED Patients Awaiting Inpatient Beds			
Q1	216 (24.3%)	355 (25.6%)	0.4189
Q2	217 (24.4%)	370 (26.6%)	
Q3	252 (28.3%)	364 (26.2%)	
Q4	205 (23.0%)	300 (21.6%)	

Association of Utilization of Ultrasound Assistance and Levels of Emergency Department Crowding.

Table 5. Crowding Measure	Q1 (Lowest) Level of Operator Experience 608 (26.6%)	Q2 Level of Operator Experience 552 (24.2%)	Q3 Level of Operator Experience 556 (24.3%)	Q4 (Highest) Level of Operator Experience 568 (24.9%)	P Value
NEDOCS					
Dangerous OC	73 (12.0%)	63 (11.4%)	62 (11.2%)	50 (8.8%)	0.3904
Severe OC	128 (21.1%)	90 (16.3%)	108 (19.4%)	121 (21.3%)	
OC	203 (33.4%)	191 (34.6%)	168 (30.2%)	184 (32.4%)	
Extremely Busy, no OC	149 (24.5%)	156 (28.3%)	164 (29.5%)	156 (27.5%)	
Busy	55 (9.0%)	52 (9.4%)	54 (9.7%)	57 (10.0%)	
Patients in the WR					
Q1	138 (23.5%)	150 (28.1%)	145 (27.0%)	147 (26.8%)	0.2550
Q2	155 (26.4%)	163 (30.5%)	145 (27.0%)	163 (29.7%)	
Q3	146 (24.9%)	116 (21.7%)	134 (24.9%)	122 (22.2%)	
Q4	148 (25.2%)	105 (19.7%)	114 (21.2%)	117 (21.3%)	
ED Occupancy					
Full	177 (29.1%)	189 (34.2%)	181 (32.6%)	188 (33.1%)	0.2669
Not Full	431 (70.9%)	363 (65.8%)	375 (67.4%)	380 (66.9%)	
# of ED Patients Awaiting Inpatient Beds (Boarders)					
Q1	153 (25.2%)	147 (26.7%)	129 (23.3%)	142 (25.0%)	0.2616
Q2	142 (23.4%)	149 (27.0%)	154 (27.8%)	142 (25.0%)	
Q3	156 (25.7%)	152 (27.6%)	148 (26.7%)	160 (28.2%)	
Q4	156 (25.7%)	103 (18.7%)	123 (22.2%)	123 (21.7%)	

Association Between Operator Level of Experience During Emergency Department Central Venous Insertions and Levels of Emergency Department Crowding.

Impact of Crowding on ED CVC

Crowding Measure	No ED CVC Insertion 7,744 (83.8%)	ED CVC Insertion 1,497 (16.2%)	P Value
NEDOCS			0.0518
Dangerous OC	965 (12.5%)	203 (13.6%)	
Severe OC	1822 (23.5%)	337 (22.5%)	
OC	2427 (31.3%)	515 (34.4%)	
Extremely Busy, no OC	1992 (25.7%)	351 (23.4%)	
Busy	538 (6.9%)	91 (6.1%)	
Patients in the WR			0.8158
Q1	1756 (23.5%)	323 (22.3%)	
Q2	1972 (26.4%)	384 (26.6%)	
Q3	1905 (25.5%)	375 (25.9%)	
Q4	1842 (24.6%)	364 (25.2%)	
ED Occupancy			0.1493
Full	2325 (30.0%)	421 (28.1%)	
Not Full	5419 (70.0%)	1076 (71.9%)	
# of ED Patients Awaiting Inpatient Beds (Boarders)			0.0795
Q1	2355 (30.5%)	406 (27.2%)	
Q2	2012 (26.0%)	411 (27.5%)	
Q3	1530 (19.8%)	315 (21.1%)	
Q4	1831 (23.7%)	362 (24.2%)	

.

Impact of Crowding on ED CVC

Table . Association Between Central Line Associated Blood Stream Infections (CLABSI) and Crowding Measures

Crowding Measure	CLABSI N=10	No CLABSI N=1,533	P Value*
NEDOCS			
Dangerous OC	1 (10%)	98 (6%)	0.8368
Severe OC	2 (20%)	311 (20 %)	
OC	4 (40%)	540 (35 %)	
Extremely Busy, no OC	2 (20%)	445 (30 %)	
Busy	1 (10%)	139 (9 %)	
Patients in the WR			
Q1	2 (20 %)	458 (30%)	0.4273
Q2	3 (30 %)	431 (28%)	
Q3	1 (10 %)	342 (22 %)	
Q4	4 (40 %)	302 (20%)	
ED Occupancy			
Full	7 (70%)	1,045 (68%)	1.0
Not Full	3 (30%)	488 (32 %)	
# of ED Patients Awaiting Inpatient Beds			
Q1	3 (30%)	379 (25%)	0.9739
Q2	3 (30%)	404 (26%)	
Q3	2 (30%)	395 (26%)	
Q4	2 (30%)	355 (23%)	

*Fisher's Exact Test
